# Supplementary material for: Efficacy of an Integrated Mobile Application System for Patients with Radiation Therapy: A Pilot Study
Source: Healthcare (Basel). 2022 Sep 5;10(9):1696. doi: 10.3390/healthcare10091696 (PMC9498518; doi:10.3390/healthcare10091696)
Supplement: Supplementary file 1 [file healthcare-10-01696-s001.zip › S1.pdf]

User questionnaire

* This questionnaire is anonymous and absolute confidentiality is guaranteed, and it is intended to know the usefulness of the radiation treatment management application (AROMA) you have used, so we would appreciate your sincere answers.

1. Where is your treatment area?

1) Head and neck 2) Thorax 3) Breast 4) Abdomen 5) pelvis 6) etc________

2. How old are you?

1) 20s 2) 30s 3) 40s 4) 50s 5) 60s 6) 70s 7) 80s 9) 90s or more

3. What is your gender?

1) Male 2) Female

4. Have you tried the radiation therapy management application, what do you think of the use of these mobile technologies in the field of radiotherapy?

1	2	3	4	5	6	7	8	9	10

I don't think it's good I think it's good

5. In general, patients have no choice but to use the treatment time to talk about discomfort or side effects during their consultation time. Are you willing to use the application to report your side effects during treatment?

1) Yes 2) No

6. Also, are you willing to use the application to communicate during follow-up with your doctor after treatment?

1) Yes 2) No

7. Your answers will be used to evaluate the usefulness of the application in the future. Would you like to know and agree to this?

1) Yes 2) No

8. Was using the application generally simple and intuitive?

1) Yes 2) No

9. Was it intuitive and easy to navigate information through the application?

1) Yes 2) No

10. How satisfied are you with the design of the app?

Size of buttons

1	2	3	4	5	6	7	8	9	10

I don't think it's good I think it's good

Configuration of the application

1	2	3	4	5	6	7	8	9	10

I don't think it's good I think it's good

Color

1	2	3	4	5	6	7	8	9	10

I don't think it's good I think it's good

Size of font

1	2	3	4	5	6	7	8	9	10

I don't think it's good I think it's good

Contents per a page

1	2	3	4	5	6	7	8	9	10

I don't think it's good I think it's good

11. 어플리케이션의 내용의 만족도는 어떻습니까?

Display treatment progress (home screen)

1	2	3	4	5	6	7	8	9	10

I don't think it's good I think it's good

Treatment schedule (Calendar)

1	2	3	4	5	6	7	8	9	10

I don't think it's good I think it's good

Management during treatment

1	2	3	4	5	6	7	8	9	10

I don't think it's good I think it's good

Report side effects during treatment

1	2	3	4	5	6	7	8	9	10

I don't think it's good I think it's good

Disease information

1	2	3	4	5	6	7	8	9	10

I don't think it's good I think it's good

12. Do you have any suggestions, ideas, or criticisms for the app?________________

____

13. What was your favorite feature? Which feature do you think you will most need?

____

14. Are there any features you would like to see added?

____

____
