# Supplementary material for: Efficacy of an Integrated Mobile Application System for Patients with Radiation Therapy: A Pilot Study
Source: Healthcare (Basel). 2022 Sep 5;10(9):1696. doi: 10.3390/healthcare10091696 (PMC9498518; doi:10.3390/healthcare10091696)
Supplement: Supplementary file 1 [file healthcare-10-01696-s001.zip › S2.pdf]

설문지

* 본 설문지는 무기명으로 절대적인 비밀을 보장하며, 사용하신 방사선 치료 관리 어플리케이션 (아로마; AROMA)의 유용성을 알기 위한 것이므로 성심 성의껏 답변해 주시면 감사하겠습니다.

1. 귀하의 치료 부위는 어디입니까?

1) 머리/목 2) 흉부 3) 유방 4) 복부 5) 골반 6) 기타________

2. 귀하의 나이는 얼마입니까?

1) 20대 2) 30대 3) 40대 4) 50대 5) 60대 6) 70대 7) 80대 9) 90 이상

3. 귀하의 성별은 무엇입니까?

1) 남 2) 여

4. 방사선 치료 관리 어플리케이션을 사용해 보시고, 이러한 모바일 기술이 방사선 치료 분야에 사용된다는 사실에 대해 어떻게 생각을 하십니까?

1	2	3	4	5	6	7	8	9	10

좋지 않은 것 같아요 좋은 것 같아요

5. 일반적으로 환자는 자신의 치료 중 불편감이나 부작용을 이야기하려면 진료시간을 이용할 수 밖에 없습니다. 치료 중 본인의 부작용 보고를 위해 어플리케이션을 사용할 용의가 있습니까?

1) 네 2) 아니요

6. 또한 치료 이후에도 의사와의 추적 관찰 중의 소통을 위해 어플리케이션을 사용할 의사가 있습니까?

1) 네 2) 아니요

7. 귀하의 답변은 추후 어플리케이션의 유용성 평가를 위해 사용될 예정입니다. 이에 대해 알고 동의하시겠습니까?

1) 네 2) 아니요

8. 어플리케이션 사용이 일반적으로 간단하고 직관적이였습니까?

1) 네 2) 아니요

9. 어플리케이션을 통한 정보 탐색이 직관적이고 쉬웠습니까?

1) 네 2) 아니요

10. 앱의 디자인에 대한 만족도는 어떻습니까?

버튼의 크기

1	2	3	4	5	6	7	8	9	10

좋지 않은 것 같아요 좋은 것 같아요

어플리케이션의 구성

1	2	3	4	5	6	7	8	9	10

좋지 않은 것 같아요 좋은 것 같아요

색깔

1	2	3	4	5	6	7	8	9	10

좋지 않은 것 같아요 좋은 것 같아요

글꼴 크기

1	2	3	4	5	6	7	8	9	10

좋지 않은 것 같아요 좋은 것 같아요

페이지당 콘텐츠

1	2	3	4	5	6	7	8	9	10

좋지 않은 것 같아요 좋은 것 같아요

11. 어플리케이션의 내용의 만족도는 어떻습니까?

치료 진행 상황 표시 (메인 화면)

1	2	3	4	5	6	7	8	9	10

좋지 않은 것 같아요 좋은 것 같아요

치료 스케줄 (달력)

1	2	3	4	5	6	7	8	9	10

좋지 않은 것 같아요 좋은 것 같아요

치료 중 관리법

1	2	3	4	5	6	7	8	9	10

좋지 않은 것 같아요 좋은 것 같아요

치료 중 부작용 보고

1	2	3	4	5	6	7	8	9	10

좋지 않은 것 같아요 좋은 것 같아요

질병 정보

1	2	3	4	5	6	7	8	9	10

좋지 않은 것 같아요 좋은 것 같아요

12. 앱에 대한 제안, 아이디어 또는 비판이 있습니까?

13. 가장 좋았던 기능은 무엇입니까? 가장 필요 없다고 생각되는 기능은 무엇입니까?

14. 추가가 됐으면 좋을 기능이 있습니까?
